# Metabolic Profiling and Functional Metabolite Distribution in Colored Tomatoes

**DOI:** 10.3390/foods14234044

**Published:** 2025-11-25

**Authors:** Ao Su, Chunxin Liu, Yurong Yang, Xudong Wang, Chengyu Wu, Dalong Li, He Zhang, Dong Liu, Xiangyang Xu, Tingting Zhao

**Affiliations:** 1 Laboratory of Genetic Breeding in Tomato, College of Horticulture and Landscape Architecture, Northeast Agricultural University, Harbin 150030, China; 2 Key Laboratory of Biology and Genetic Improvement of Horticultural Crops (Northeast Region), Ministry of Agriculture and Rural Affairs, Northeast Agricultural University, Harbin 150030, China; 3 College of Plant Protection, Northeast Agricultural University, Harbin 150030, China

**Keywords:** tomato color, wide-targeted metabolomics, functional metabolites, distribution patterns

## Abstract

Tomato (*Solanum lycopersicum* L.) is valued for its versatile consumption and rich nutrients. Current research on functional metabolite distribution in tomatoes mostly focuses on a few varieties, limiting comprehensive understanding across different colored types. This study investigated metabolic characteristics and functional metabolite distribution in colored tomatoes via metabolomic analysis and functional metabolite quantification across diverse germplasm resources. Metabolomic analysis identified 910 metabolites from four colored cherry tomato varieties. Significantly differential metabolite analysis revealed most were flavonoids (27 in total) and alkaloids (14 in total). Additionally, KEGG enrichment analysis identified 4 significantly enriched pathways, mainly related to amino acid biosynthesis, degradation, and metabolism. Quantification across 113 tomato germplasm resources revealed that red tomatoes had higher lycopene; brown tomatoes were rich in lycopene, chlorophyll, and β-carotene; green tomatoes contained higher chlorophyll, saponin, vitamin E, and naringenin. This study provides an important reference for consumers to select colored tomatoes and for breeders to conduct targeted genetic improvement.

## 1. Introduction

Fruit color is a key trait of interest to breeders and consumers, and tomato (*Solanum lycopersicum* L.) is a widely popular vegetable due to its diverse fruit colors, unique flavor, abundant nutrients, and functional metabolites that confer health benefits. Tomato fruit color is critical to consumers’ perception of quality—an aesthetic parameter linked to better flavor, nutrition, and freshness [1]—and is determined by carotenoids, flavonoids, and chlorophyll: red fruits accumulate more lycopene, green fruits more chlorophyll, yellow fruits more yellow carotenoids, and brown fruits come from a mutant of red-fruited tomatoes, in which chlorophyll is difficult to degrade [2,3,4].

Over the past few decades, many studies have pointed out that there is a direct relationship between the intake of bioactive compounds in tomatoes and the prevention of diseases as well as the promotion of human health [5]. These bioactive components include phytochemicals such as carotenoids and polyphenols [5,6]. Specifically, lycopene aids atherosclerosis management [7,8], phenolic compounds mitigate cardiovascular diseases and certain cancers [9,10], β-carotene supports vision [11,12,13], alkaloids and saponins enhance immunity [14,15,16,17], chlorophyll inhibits cancer by binding mutagens [18,19], and vitamins C and E exert synergistic antioxidant effects (free radical scavenging, cell membrane protection) [20,21,22]. These phytochemicals act synergistically in natural matrices, conferring stronger health benefits than single. Therefore, ingestion of these phytochemicals in their natural matrix seems to be more efficient than ingesting one of them alone or in dietary supplements [23].

In recent years, widely targeted metabolomics based on the ultra-high-performance liquid chromatography coupled to triple-quadrupole mass spectrometry (UHPLC-QqQ-MS) technique has been widely used in the study of vegetable metabolism as an effective detection method [24,25]. Wang et al. clarified the key pigments underlying pepper color phenotypes (delphinidin-type anthocyanins for purple, capsanthin for red) and their potential regulatory genes using integrated transcriptomic and metabolomic approaches [26]. Mao et al. analyzed the peel, outer yellow flesh (LR), inner red flesh (HR), and core of *Actinidia chinensis* cv. Hongyang via widely targeted metabolomics, and revealed that anthocyanins (e.g., cyanidin-3,5-O-diglucoside) and quercetin-3-O-glucoside were key substances underlying the flesh color differences [27]. Jia et al. generated tomato mutants of nine transcription factors via CRISPR/Cas9 technology, and integrated metabolomic and transcriptomic analyses revealed that different transcription factors regulate carotenoid accumulation through ethylene-dependent or -independent pathways, leading to diverse fruit color phenotypes [28].

Here, we conducted a widely targeted metabolomic analysis of four different colored tomatoes using this technique and determined some important functional metabolites in 113 tomato germplasm materials. This study aims to reveal the distribution pattern of important functional metabolites in tomato fruits of different color types. In general, this study not only provides a good reference for people to choose different colored tomatoes according to different nutrient requirements, but also offers a valuable basis for breeders to develop breeding programs.

## 2. Materials and Methods

### 2.1. Plant Materials

**Figure 1:**
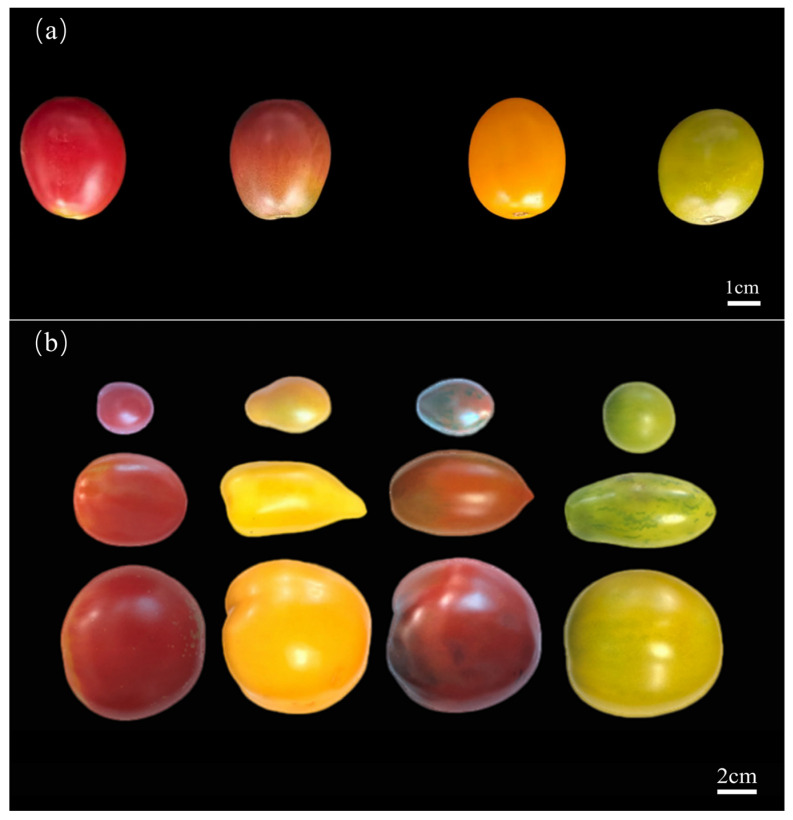
Four colors of cherry tomatoes for metabolome analysis. (**a**): Four different colors of cherry tomatoes were used for metabolome sequencing (*n* = 3). (**b**): A total of 113 tomato germplasm resources were collected, which included 12 different types.

Four different colors of cherry tomatoes (red, brown, yellow, and green) with similar maturity periods were used for metabolome sequencing. Three biological replicates were included for each color of tomato, and their fruit morphology is shown in Figure 1a. A total of 113 tomato germplasm materials with different types (big, roman, and cherry) and colors (70 red, 19 yellow, 14 green, and 10 brown)—provided by the Tomato Genetics and Breeding Team of Northeast Agricultural University in Harbin, China—were used to determine important functional metabolites, and their fruit morphology is shown in Figure 1b. All these materials were grown in the greenhouse (14-h light: 10-h dark cycles; day temperature: 25 °C; night temperature: 20 °C; 60% relative humidity) of the Horticulture Experimental Station of Northeast Agricultural University.

### 2.2. Metabolite Extraction

Four colors of cherry tomatoes were included in this study, with three biological replicates per color (12 samples in total). All samples were lyophilized under vacuum for 48 h, and the lyophilized samples were crushed with a grinder at 40 Hz for 180 s. A 50 mg aliquot of each sample was precisely weighed and transferred to an Eppendorf tube, followed by the addition of 700 μL of extraction solution [methanol: water (3:1, *v*/*v*), precooled at −40 °C, containing an internal standard]. After vortexing for 30 s, the samples were homogenized at 35 Hz for 4 min and sonicated for 5 min in an ice-water bath; this homogenization–sonication cycle was repeated 3 times. The samples were then shaken overnight at 4 °C on a shaker for metabolite extraction. The samples were centrifuged at 12,000 rpm (relative centrifugal force, RCF = 13,800× *g*; rotor radius, R = 8.6 cm) for 15 min at 4 °C. Subsequently, the supernatant was carefully filtered through a 0.22-μm microporous membrane, diluted 15-fold with a methanol:water mixture (3:1, *v*/*v*, containing an internal standard), vortexed for 30 s, and transferred to 2 mL glass vials. These vials were stored at −80 °C until UHPLC-MS analysis.

### 2.3. UHPLC–MS

The UHPLC separation was carried out using an ExionLC System (SCIEX, Framingham, MA, USA). Mobile phase A was 0.1% formic acid in water, and mobile phase B was acetonitrile. Chromatographic separation of the target compounds was achieved using a Waters UPLC column (Acquity UPLC HSS T3 1.8 μm 2.1 × 100 mm) (Waters, MA, USA). The column temperature was set at 4 °C, the injection volume is 2 μL, and the flow rate is 400 μL /min. A Sciex QTrap 6500+ (SCIEX, Framingham, MA, USA) was applied for assay development. Typical ion source parameters were as follows: IonSpray Voltage: +5500/−4500 V; Curtain Gas: 35 psi; Temperature: 400 °C; Ion Source Gas 1: 60 psi; Ion Source Gas 2: 60 psi; DP: ±100 V.

### 2.4. Statistical Analysis

All MS data acquisition and quantitative analysis of target compounds were performed using the SCIEX Analyst Work Station Software (Version 1.6.3). The MSConverter software (Version 3.0) was used to convert the original mass spectrometry data into TXT format. R packages (Version 1.1.4) and a self-built database were applied to peak detection and annotation. Standard data from 12 samples were analyzed using principal component analysis (PCA) and orthogonal partial least squares discriminant analysis (OPLS-DA) via SIMCA software (V16.0.2, Sartorius Stedim Data Analytics AB, Umeå, Sweden). A permutation test (200 permutations) was performed to avoid overfitting of the OPLS-DA model. Variable importance in projection (VIP) values of all metabolites from the OPLS-DA were calculated using the first component. Differential metabolites were screened based on the following criteria: (i) VIP > 1 in pairwise comparisons; (ii) *p*-value < 0.05. The *p*-values were calculated using an independent two-sample *t*-test. One-way analysis of variance (one-way ANOVA) tested overall significance of differences in functional metabolites among 113 tomato samples, followed by Tukey’s test for pairwise group comparisons with multiple comparison correction; *p* < 0.05 was considered significant. The Pearson correlation test was performed using SPSS (Statistical Product and Service Solutions, Version 25.0).

### 2.5. Kyoto Encyclopedia of Genes and Genomes (KEGG) Annotations and Metabolic Pathway Analyses of Differential Metabolites

Differential metabolites were annotated and classified using the Kyoto Encyclopedia of Genes and Genomes (KEGG) database (http://www.kegg.jp/kegg/pathway.html, accessed on 25 June 2023). Through comprehensive analysis of the pathways where the differential metabolites were located (including enrichment analysis and topological analysis), we screened and identified the key pathways.

### 2.6. Method for Measurement of Lycopene, Beta-Carotene and Chlorophyll Content in 113 Tomato Germplasm Materials

Lycopene in 0.001 g fresh fruit samples was dissolved in distilled water, vortexed while incubated in a water bath at 30 °C for 1 h, and then extracted using a hexane:ethanol:acetone (2:1:1, *v*/*v*) mixture following the method described by Theeranat Suwanaruang [29]. Absorbance of samples determined at 503 nm by spectrophotometry.

Lycopene (ug/g) = A503 nm × 137.4(1)

The extraction method of β-carotene was performed with reference to Daood, H.G. et al. [30]. Twenty milligrams of β-carotene standard (B21973, Yuanye Bio-Technology Co., Ltd., Shanghai, China) was dissolved in 25 mL dichloromethane, and then diluted with the mobile phase (80% methanol, 17% isopropanol, and 3% dichloromethane, *v*/*v*) to obtain concentrations of 0.5 mg/mL, 0.25 mg/mL, 0.125 mg/mL, 0.0625 mg/mL, and 0.01 mg/mL, respectively, for the preparation of the standard curve. 20 µL of each standard solution and 20 µL of each sample (after being filtered through a 0.22-μm Teflon (PTFE) syringe filter) were injected onto the HPLC system. An Agilent 1260 HPLC system (Palo Alto, CA, USA) was equipped with a C18 column (250 mm × 4.6 mm, 5 μm; Waters Symmetry).

The chlorophyll content of tomato fruits was determined by a spectrophotometer. Tomato fruit sample was ground with 2 mL of 95% ethanol in a mortar. The homogenate was transferred to a centrifuge tube. The mortar was washed twice with 95% ethanol, and the washing liquid was also transferred to the same centrifuge tube. The volume in the centrifuge tube was then adjusted to a final volume of 4 mL with 95% ethanol. After centrifugation at 7000 rpm for 8 min, the supernatant was carefully aspirated to determine the absorbance at 649 nm and 665 nm.

Chlorophyll A content (mg/g FW) = [(13.95A_665_ − 6.88A_649_) × volume of extraction liquid × dilution ratio]/fresh weight of sample (2)

Chlorophyll B content (mg/g FW) = [(24.96A_649_ − 7.32A_665_) × volume of extraction liquid × dilution ratio]/fresh weight of sample (3)

Total chlorophyll content (mg/g FW) = chlorophyll A content + chlorophyll B content(4)

### 2.7. Determination of Vitamin C, Vitamin E, Alkaloids, Total Saponins, and Five Phenols in 113 Tomato Germplasm Materials

The content of vitamin C was determined using a kit (G0201W, Grace Biotechnology, Suzhou, China) following the instructions. Similarly, the content of vitamin E was determined using a kit (VE-1-G, Comin, Suzhou, China) by the colorimetric method, following the instructions. Alkaloids were measured using a kit (SWJ-2-Y, Comin, Suzhou, China) by visible spectrophotometry. The total saponin content was determined using a kit (TSG-1-Y, Comin, Suzhou, China) by the colorimetric method. The extraction method of phenolic substances was as follows: After homogenization, the tomato sample was dried at low temperature for 36 h and ground into powder. Four milliliters (4 mL) of methanol was added to 0.2 g of tomato powder. The mixture was then extracted at 25 °C for 1 h with continuous shaking. After centrifugation at 8000 rpm for 10 min, the supernatant was filtered through a 0.22 μm filter membrane. Standards of caffeic acid, ferulic acid, rutin, naringin, and cinnamic acid (Yuanye Bio-Technology Co., Ltd., Shanghai, China) were dissolved in methanol and prepared into standard solutions with concentrations of 0.2 mg/mL, 0.1 mg/mL, 0.05 mg/mL, 0.025 mg/mL, and 0.012 mg/mL for the standard curve. The equipment used for these experiments was the same as mentioned above. The mobile phase A was methanol, and mobile phase B was 1% (*v*/*v*) acetic acid aqueous solution.

## 3. Results

### 3.1. PCA, OPLS-DA Analysis

**Figure 2:**
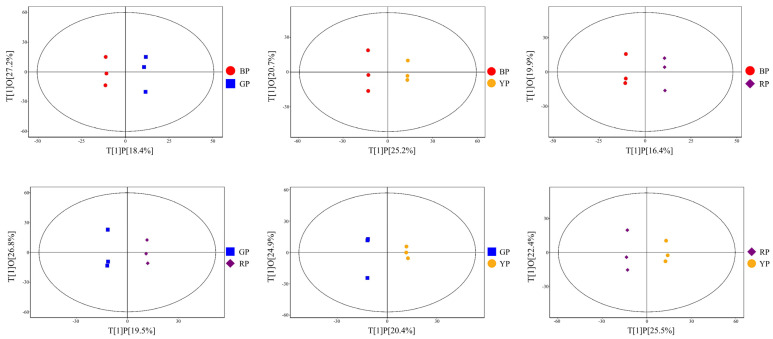
The OPLS-DA score plots of pairwise comparison of four tomato color groups. BP: brown group; GP: green group; RP: red group; YP: yellow group.

Based on the self-built local tomato metabolite database, 910 metabolites were identified in tomato samples via qualitative and quantitative analyses. Prior to multivariate statistical analysis, all metabolite data were normalized by total ion current (TIC) scaling, and repeated analyses with TIC-normalized data. To compare metabolite profiles of four color tomato groups (BP, GP, RP, YP) and assess data quality, PCA was performed on 12 samples plus quality control (QC) samples. As shown in Figure S1, PC1 and PC2 explained 19.9% and 10.3% of the variance, respectively. The four color groups showed no clear separation via this unsupervised method, while QC samples clustered tightly, confirming data reliability. Subsequently, supervised OPLS-DA was applied to identify inter-group differences. In Figure 2, samples from different color groups were well separated along t[1]P (predictive component, 16.4–25.5% variance explained) and t[1]O (orthogonal component, 19.9–27.2% variance explained). OPLS-DA models exhibited strong performance, with R^2^X (0.364–0.479), R^2^Y (0.999–1.000), and Q^2^ (0.429–0.677). Subsequently, the possibility of sample overfitting was ruled out through the OPLS-DA permutation test (Figure S2). The above results indicate that there are significant differences in metabolites among tomato samples of different colors.

### 3.2. Differential Metabolite Analysis

**Figure 3:**
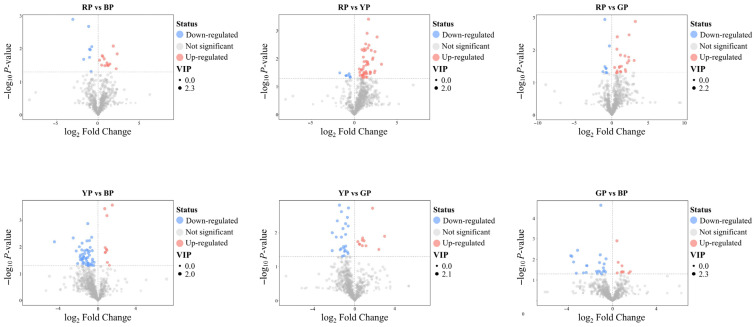
Volcano plots display the upregulated, downregulated, and insignificant metabolites. BP, brown group. GP, green group. RP, red group. YP, yellow group.

Based on the thresholds of VIP > 1 and *p* < 0.05, metabolites with significant differences were screened out (Figure 3) through statistical analysis. In the comparison of red and yellow, there were 48 significantly up-regulated and 8 significantly down-regulated metabolites; in the comparison of red and green, 21 significantly up-regulated and 8 significantly down-regulated metabolites; in the comparison of red and brown, 14 significantly up-regulated and 8 significantly down-regulated metabolites; in the comparison of yellow and green, 11 significantly up-regulated and 24 significantly down-regulated metabolites; in the comparison of yellow and brown, 10 significantly up-regulated and 50 significantly down-regulated metabolites; and in the comparison of green and brown, 9 significantly up-regulated and 23 significantly down-regulated metabolites. Details of these metabolites are shown in File S1.

#### 3.2.1. Main Differential Metabolites and Their Content Distribution in Different Colored Tomatoes

Further analysis indicated that flavonoids and alkaloids were the dominant significantly differentially expressed metabolites across all pairwise comparisons (File S1). Their ratios relative to total significant differential metabolites were as follows: Red vs. Yellow (5/14), Red vs. Green (8/29), Red vs. Brown (5/22), Yellow vs. Green (2/7), Yellow vs. Brown (4/15), and Green vs. Brown (5/16).

Metabolomics analysis identified 122 flavonoids and 91 alkaloids in total (File S2). For significant differential flavonoids: Red and brown fruits had higher levels than green and yellow fruits, with yellow fruits showing the lowest levels among the four color groups. Only two differential flavonoids were detected between red and brown fruits—Alpha-Toxicarol (Red > Brown) and schaftoside (Red < Brown) (File S3).

For significant differential alkaloids: Most were more abundant in red fruits than yellow, green, and brown fruits, except rutacridone and riddelline (Red < Yellow). In pairwise comparisons: Yellow vs. Brown (synephrine up-regulated, 5 others down-regulated); Yellow vs. Green (4 down-regulated, 3 up-regulated); Green vs. Brown (2 up-regulated, 2 down-regulated) (File S3).

#### 3.2.2. Common Differential Metabolites and Their Content in Different Colored Tomatoes

Furthermore, by analyzing the common significantly differentially expressed metabolites in different comparison groups, we found that koenigicine and neocnidilide were most abundant in red fruits; proline betaine peaked in yellow fruits; citreorosein was highest in brown fruits and higher in green than yellow fruits; and (s)-n-methylcoclaurine showed higher levels in red and brown fruits than in green fruits, with the lowest content in yellow fruits. Detailed information is provided in File S3.

### 3.3. Metabolic Pathway Analysis of Differential Metabolites

**Figure 4:**
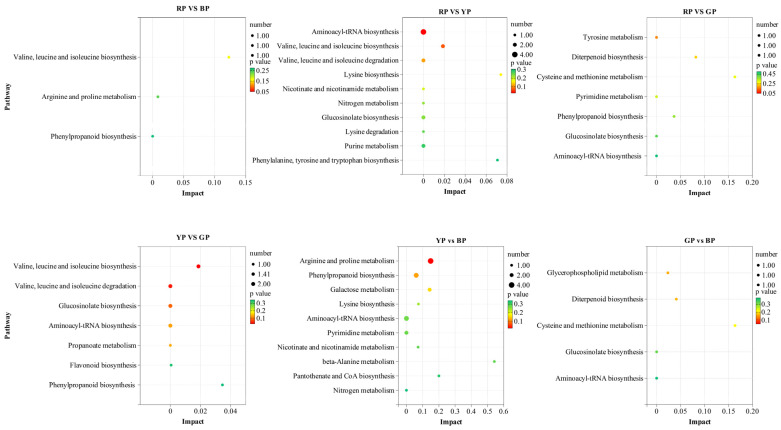
KEGG enrichment analysis of the DAMs. BP, brown group. GP, green group. RP, red group. YP, yellow group.

In this study, KEGG significant enrichment results of differential metabolites screened based on VIP > 1 and *p* < 0.05 were shown in Figure 4 (detailed pathways in File S4). Red vs. Yellow comparisons identified 14 enriched pathways (1 significantly enriched), where L-glutamine, L-lysine, L-isoleucine, and L-leucine were significantly enriched in the aminoacyl-tRNA biosynthesis pathway; Red vs. Green and Red vs. Brown comparisons yielded 7 and 3 enriched pathways, respectively, with no significant enrichment; Yellow vs. Green comparisons resulted in 7 enriched pathways (2 significantly enriched), with L-leucine and L-isoleucine co-enriched in both the valine, leucine and isoleucine biosynthesis pathway and valine, leucine and isoleucine degradation pathway; Yellow vs. Brown comparisons showed 19 enriched pathways (1 significantly enriched), where L-citrulline, L-arginine, n-acetyl-l-glutamate 5-semialdehyde, and L-glutamine were significantly enriched in the arginine and proline metabolism pathway; Green vs. Brown comparisons identified 5 enriched pathways with no significant enrichment. Notably, all significantly enriched pathways were associated with amino acid biosynthesis, degradation, or metabolism, and amino acids enriched in these pathways (e.g., L-glutamine, L-lysine, L-isoleucine, L-leucine) exhibited significantly higher levels in yellow tomatoes than in red, green, and brown tomatoes (File S5).

### 3.4. The Composition Rule of Important Functional Metabolites in Tomatoes of Four Colors

**Figure 5:**
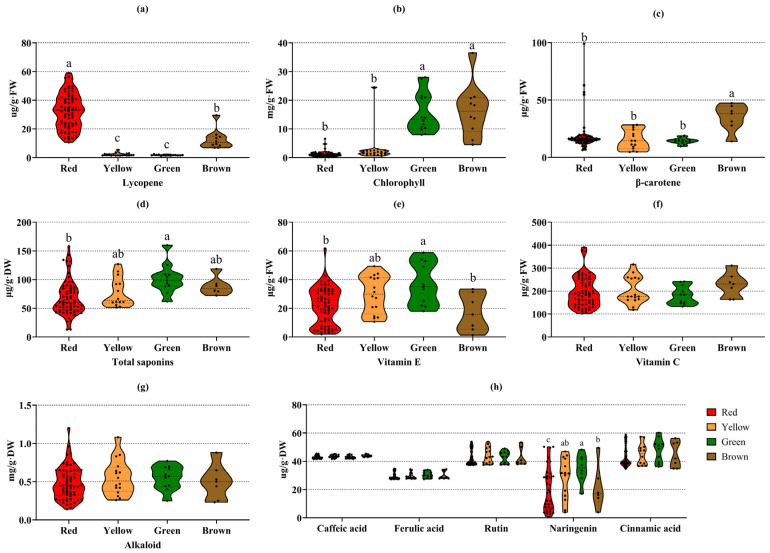
The distribution patterns of seven important functional metabolites in tomato varieties of different colors. (**a**) Lycopene; (**b**) Chlorophyll; (**c**) β-carotene; (**d**) Total saponins; (**e**) Vitamin E; (**f**) Vitamin C; (**g**) Alkaloid; (**h**) Phenols. A total of 70 red tomato samples, 19 yellow tomato samples, 14 green tomato samples, and 10 brown tomato samples were analyzed. Different lowercase letters indicate significant differences among groups (*p* < 0.05), and the same letter indicates no significant difference.

Many studies have reported the close relationship between fruit color and metabolites [31,32,33,34,35]. In tomato, Dono et al. obtained San Marzano (SM) tomato mutants with yellow, brown and pink fruits by constructing introgression lines (IL), aiming to explore the differences in metabolite composition in different colored tomatoes with the same genetic background [36]. However, this is not representative of tomato materials with different genetic backgrounds. Here, we identified several functional metabolites from 113 tomato germplasm materials with different shapes and genetic backgrounds by HPLC and spectrophotometry, in order to find the composition rule of these important metabolites in different colored tomatoes (Figure 5).

For lycopene (Figure 5a), the average content followed the order of red > brown > yellow > green (distribution ranges: 12.88–49.93, 6.88–16.1, 1.44–3.75, 1.21–2.46, respectively); red tomatoes had significantly higher levels than brown, yellow, and green tomatoes, brown tomatoes were significantly higher than green and yellow ones, while green and yellow tomatoes showed extremely low and non-significant differences. Chlorophyll (Figure 5b) exhibited an average content pattern of brown > green > yellow > red (13.6–21.2, 8–14.3, 0.62–2.95, 0.14–1.96, respectively), with no significant difference between brown and green tomatoes—both were significantly higher than yellow and red tomatoes, which had extremely low and non-significant levels. For β-carotene (Figure 5c), the average content was brown > red > yellow > green (27.72–47.3, 6.39–25.86, 4.72–28.38, 9.6–18.92, respectively), with brown tomatoes showing significantly higher levels than the other three colors and no significant differences among red, yellow, and green tomatoes. Total saponin (Figure 5d) followed green > brown > yellow > red (88.2–108.78, 72.34–92.65, 51.49–66.32, 39.31–63.18, respectively), with green tomatoes significantly higher than red ones and no significant differences among other groups. Vitamin E (Figure 5e) exhibited an average content order of green > yellow > red > brown (17.91–58.81, 10.64–49.36, 1.91–48.81, 1.27–33.4, respectively), with green tomatoes significantly higher than red and brown ones and no significant differences in other comparisons. For naringenin (Figure 5f–h), the average content in different colored tomatoes followed green > yellow > brown > red (27.85–48.25, 30.24–46.95, 14.1–17.81, 0.86–12.81, respectively), with green tomatoes significantly higher than brown and red ones, and yellow tomatoes significantly higher than red ones. In contrast, vitamin C, alkaloids, rutin, caffeic acid, ferulic acid, and cinnamic acid (Figure 5f–h) showed no significant content differences among different colored tomatoes.

### 3.5. Correlation Among Seven Functional Metabolites in Tomatoes of Four Colors

**Figure 6:**
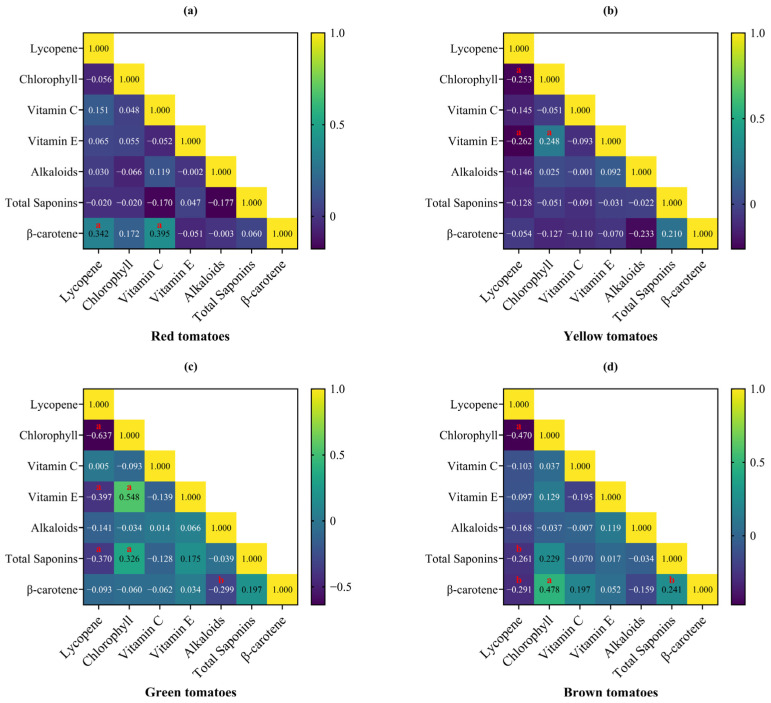
Pearson’s correlation analysis among seven functional metabolites in different color tomatoes. (**a**) Red tomatoes; (**b**) Yellow tomatoes; (**c**) Green tomatoes; (**d**) Brown tomatoes. A total of 70 red tomato samples, 19 yellow tomato samples, 14 green tomato samples, and 10 brown tomato samples were analyzed. Different lowercase letters indicate significant differences among groups (*p* < 0.05), and the same letter indicates no significant difference.

Pearson correlation (r) analysis results are shown in Figure 6. In red tomatoes, β-carotene was significantly positively correlated with lycopene and vitamin C (Figure 6a). In yellow tomatoes, lycopene was significantly negatively correlated with chlorophyll and vitamin E, while chlorophyll was significantly positively correlated with vitamin E (Figure 6b). In green tomatoes, β-carotene was significantly negatively correlated with alkaloids; lycopene was not only significantly negatively correlated with chlorophyll and vitamin E, but also significantly negatively correlated with total saponins. Moreover, chlorophyll in green tomatoes was positively correlated with total saponins in addition to vitamin E (Figure 6c). Compared with green tomatoes, lycopene in brown tomatoes was not only significantly negatively correlated with chlorophyll and total saponins, but also negatively correlated with β-carotene. In contrast to red tomatoes, β-carotene in brown tomatoes was negatively correlated with lycopene, while it was significantly positively correlated with chlorophyll and total saponins (Figure 6d).

## 4. Discussion

Many studies have shown that flavonoids are closely related to the color of tomato fruits [37,38]. Through metabolomics, Liu, Y. et al. found that there are 188 kinds of flavonoids in the fruits of four different colors of pepper, among which the content of anthocyanins, flavonoids, and flavonols in purple varieties was significantly higher than that in other colors of pepper [32]. Through metabolomic analysis, Yang et al. found that the content of flavonoids in red *Nitraria* L. fruits was significantly higher than that in orange fruits [39]. In this study, a total of 122 flavonoids were also identified. Additionally, a high proportion of flavonoids was observed among the significantly differentially expressed metabolites. It is noteworthy that the content of significantly differentially expressed flavonoids in red and brown fruits is higher than that in yellow and green fruits, while the content of significantly differentially expressed flavonoids in yellow fruits is lower than that in red, green and brown fruits. Furthermore, we found that the content of koenigicine and neocnidilide were the highest in red fruits. The content of proline betaine was the highest in yellow fruits. The content of citreorosein was the highest in brown fruits. These results indicate that the differentiated accumulation of these flavonoids and non-flavonoids is closely related to the color phenotype of tomato fruits.

Amino acid-derived compounds are important components that cause aroma. In addition, lipid-derived compounds, phenolic derivatives, as well as monoterpenoids and sesquiterpenoids can also produce aroma [40,41]. Co-incubation of exogenous L-phenylalanine, L-leucine, L-isoleucine and L-valine with melon pulp respectively promotes the production of corresponding aromatic amino acid derivatives [42]. Through KEGG analysis in this experiment, it was found that the significantly enriched pathways in different comparison groups were related to the biosynthesis, degradation, and metabolism of amino acids. Notably, the content of amino acids (e.g., L-glutamine, L-lysine, L-isoleucine, and L-leucine) in these significantly enriched pathways was significantly higher in yellow tomatoes than in red, green, and brown tomatoes. These results indicate that yellow tomatoes may produce more amino acid derivatives and thus more unique fragrances. In the future, we will further explore whether these amino acids play an important role in the aroma regulation of different colored tomatoes.

Flores, P. et al. determined the contents of lycopene, β-carotene and chlorophyll in 53 tomato materials of different genotypes across 6 tomato color groups [43]. They found that the contents of lycopene and β-carotene in red and brown tomato fruits were not significantly different, but were both significantly higher than those in yellow tomatoes. The chlorophyll content was the highest in brown fruits and was slightly higher in yellow tomato fruits than in red tomatoes. In this study, except that the lycopene content differed from that in Flores et al.’s research, the differences in chlorophyll and β-carotene among different colored tomatoes were basically consistent with the results [43]. Faller et al. demonstrated that phenols possess multiple physiological activities and exert certain effects on the quality and flavor of tomatoes [44]. Chang et al. found that the average content of naringenin in cherry tomatoes of different colors was brown > green > yellow > red [45]. In contrast, this study measured tomatoes of different shapes and colors, and found that the distribution pattern of the average naringenin content in tomatoes of different colors was green > yellow >brown >red. We believe that this study utilized tomato germplasm resources of different shapes and a broader range, which directly led to differences in the accumulation patterns of carotenoids (lycopene) and phenols (naringenin) compared with the aforementioned studies.

Lycopene has strong antioxidant capacity due to its high content of conjugated double bonds [46]. Studies have demonstrated that lycopene can exert a synergistic antioxidant effect with other antioxidant substances such as β-carotene, vitamin C, and vitamin E, thereby enhancing overall antioxidant capacity [47]. This study found that in red tomatoes, β-carotene was significantly positively correlated with lycopene and vitamin C. This significant positive correlation may further strengthen the synergistic antioxidant effect among the three substances, indicating that red tomatoes may possess stronger antioxidant capacity.

## 5. Conclusions

**Figure 7:**
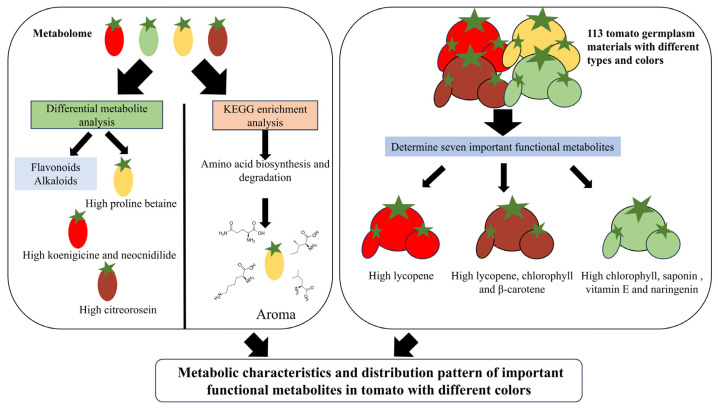
Distribution pattern of important functional metabolites in tomato fruits of different color types.

The framework of this research is illustrated in Figure 7. Metabolomic and quantitative analyses clarified the metabolic uniqueness of different colored tomatoes, with flavonoids and alkaloids as key differential metabolites and amino acid biosynthesis, degradation, and metabolism emerging as core enriched biological processes. Yellow tomatoes accumulated high levels of L-glutamine, L-lysine, L-isoleucine, and L-leucine, and tomatoes of different colors showed significant differences in the content of functional metabolites. Practically, these insights refine germplasm selection for breeders by pinpointing color-linked metabolic pathways and target metabolites for nutritional and quality trait improvement. For the food industry, they can guide the development of functional tomato products by leveraging color-specific metabolite advantages. Consumers gain a more precise basis for color-based selection, aligning dietary needs with specific functional components.

## Data Availability

The original contributions presented in this study are included in the article/Supplementary Material. Further inquiries can be directed to the corresponding author.

## Supplementary Materials

The following supporting information can be downloaded at: https://www.mdpi.com/article/10.3390/foods14234044/s1, Figure S1: Principal component analysis (PCA) plot in four color groups of cherry tomato.; Figure S2: OPLS-DA permutation test of pairwise comparison of four cherry tomato color groups; File S1: Significantly differentially expressed metabolites; File S2: All flavonoids and alkaloids identified through metabolome analysis; File S3: Significantly differentially expressed flavonoids and alkaloids and common significantly differentially expressed metabolites; File S4: KEGG significant enrichment analysis; File S5: The expression differences of the amino acids annotated in the KEGG significantly enriched pathway in tomatoes of different colors.

## Abbreviations

The following abbreviations are used in this manuscript:

UHPLC-QqQ-MS	Ultra-high-performance liquid chromatography coupled to triple-quadrupole mass spectrometry
VIP	Variable importance in projection
KEGG	Kyoto Encyclopedia of Genes and Fenomes
PCA	Principal component analysis
OPLS-DA	Orthogonal partial least squares discriminant analysis
SM	San Marzano
IL	Introgression lines
