# Supplementary material for: Metabolic Profiling and Functional Metabolite Distribution in Colored Tomatoes
Source: Foods. 2025 Nov 25;14(23):4044. doi: 10.3390/foods14234044 (PMC12692197; doi:10.3390/foods14234044)
Supplement: Supplementary file 1 [file foods-14-04044-s001.zip › foods-3967464-supplementary.pdf]

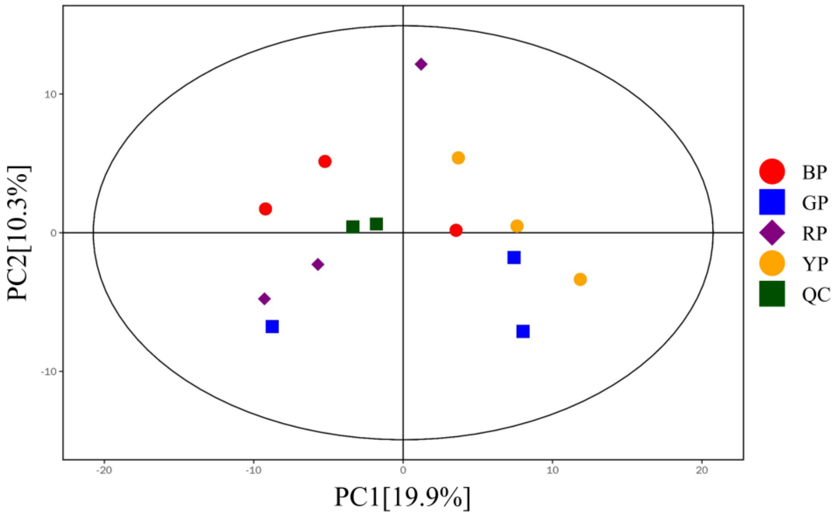

Figure. S1 Principal component analysis (PCA) plot in four color groups of cherry tomato. BP: brown group. GP: green group. RP: red group. YP: yellow group. QC: Quality control samples. PC1 and PC2 represent the scores of the first and second ranked principal components, respectively.

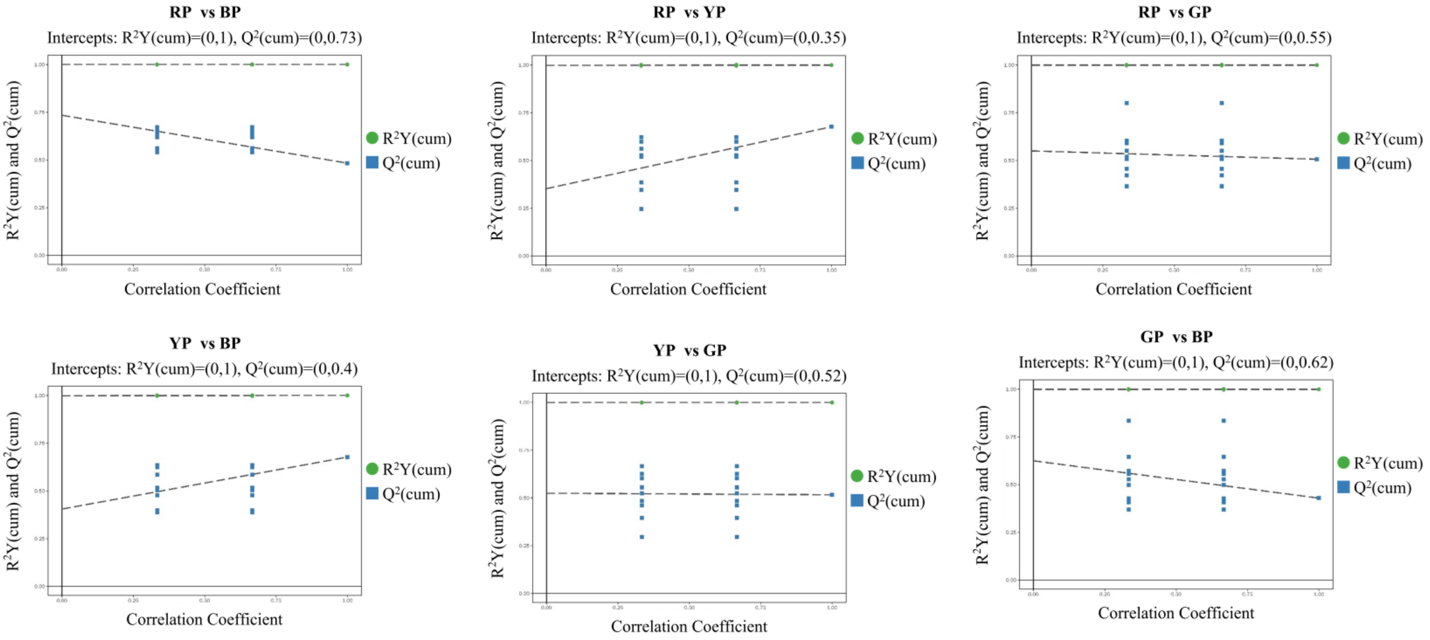

Figure. S2 OPLS-DA permutation test of pairwise comparison of four cherry tomato color groups. BP: brown group. GP: green group. RP: red group. YP: yellow group.

File S1: Significantly differentially expressed metabolites;

File S2: All flavonoids and alkaloids identified through metabolome analysis;

File S3: Significantly differentially expressed flavonoids and alkaloids and common significantly differentially expressed metabolites;

File S4: KEGG significant enrichment analysis;

File S5: The expression differences of the amino acids annotated in the KEGG significantly enriched pathway in tomatoes of different colors.
